# Epstein–Barr Virus–host epigenetic interplay: mechanisms of regulation and therapeutic potential

**DOI:** 10.1186/s13148-026-02181-2

**Published:** 2026-07-06

**Authors:** Sanaz Baghban Rahimi, Ahmad Hosseinzadeh Adli, Hossein Bannazadeh Baghi, Vahdat Poortahmasebi, Jamal Sarvari

**Affiliations:** 1 Department of Bacteriology and Virology, School of Medicine, Shiraz University of Medical Sciences, Shiraz, Iran; 2 Department of Microbiology and Virology, Faculty of Medicine, Tabriz University of Medical Sciences, Tabriz, Iran; 3 Infectious and Tropical Diseases Research Center, Tabriz University of Medical Sciences, Tabriz, Iran; 4 Immunology Research Center, Tabriz University of Medical Sciences, Tabriz, Iran; 5 Research Center of Clinical Virology, Tehran University of Medical Sciences, Tehran, Iran; 6 Gastroenterohepatology Research Center, Shiraz University of Medical Sciences, Shiraz, Iran

**Keywords:** Epstein–Barr virus, Epigenetic, Therapeutic potential

## Abstract

The Epstein-Barr virus (EBV) has evolved complex mechanisms to manipulate the host immune system and epigenetic machinery, enabling it to establish lifelong latent infection within host cells. Emerging evidence strongly indicates that the EBV acts as a main epigenetic manipulator by silencing host regulatory genes, altering host chromatin landscapes and exploiting B-cell biology. Key mechanisms include viral-mediated recruitment of host DNA methyltransferases and histone-modifying enzymes; modulation of host non-coding RNAs and chromatin architectural factors; and targeted disruption of interferon signaling through RNA methylation and restructuring of host chromatin. These integrated processes allow EBV to maintain a lifelong infection in humans, evade both innate and adaptive immunity, and respond to reactivation signals. Understanding the virus-host epigenetic interplay offers new opportunities for therapeutic interventions targeting epigenetic regulators in EBV-associated diseases.

## Introduction

The Epstein–Barr virus (EBV), also known as human herpesvirus 4 (HHV-4), belongs to the family Orthoherpesviridae and the genus Lymphocryptovirus, with a genome of approximately 171,823 bp. Two EBV types circulate in populations worldwide, but type 1 is the most prevalent one globally, particularly in developed nations [1]. EBV types 1 and 2 differ mainly in the EBV nuclear antigen (EBNA) gene family, including EBNA1, EBNA2, EBNA3A, EBNA3B, EBNA3C, and EBNA-LP, which are essential for B-cell immortalization [2]. Sequence variations in the EBNA2 and EBNA3 proteins alter their interactions with host proteins and target gene promoters, leading to differential gene regulation and the distinct biological properties of the two EBV types [3].

EBV has been reported to be associated with a wide spectrum of human diseases, ranging from asymptomatic, self-limiting infectious illnesses to different malignancies and autoimmune disorders [4]. Primary EBV infection is often asymptomatic in childhood; however, it could cause a more severe and symptomatic illness in adolescence known as infectious mononucleosis or glandular fever [5]. Moreover, EBV, as an oncogenic virus, can evade immune system surveillance, establishing persistent latent infection in host cells that may lead to malignancies [6], such as Burkitt lymphoma (BL), Hodgkin lymphoma (HL), post-transplant lymphoproliferative disorder (PTLD), diffuse large B-cell lymphoma (DLBCL), extranodal NK/T-cell lymphoma, nasopharyngeal carcinoma (NPC), and gastric cancer (GC) [7]. Persistent latent EBV infection may also be associated with autoimmune diseases, including multiple sclerosis (MS) [8, 9], systemiclupus erythematosus (SLE), rheumatoid arthritis (RA), Sjögren’s syndrome, inflammatory bowel disease, and type 1 diabetes [10].

EBV gene expression states are extensively regulated by epigenetic mechanisms, including DNA methylation, histone modifications, and chromatin remodeling [11]. EBV actively manipulates the epigenetic landscape of host cells to evade the immune system and, in many cases, promotes the development of EBV-associated cancers. Epigenetic regulation is central to EBV’s ability to maintain long-term latency, manipulate host gene expression, and evade immune surveillance [6]. By remodeling chromatin, modulating histone modifications, and utilizing viral non-coding RNAs, EBV suppresses tumor suppressor genes and immune regulators, creating a permissive environment for viral persistence and reactivation [12]. Understanding these virus-host epigenetic interactions provides promising avenues for targeted therapies in EBV-associated diseases.

## Virological features

### Virus structure

EBV has a toroid-shaped DNA core within a nucleocapsid containing 162 capsomeres, an outer envelope with glycoprotein spikes, and a protein tegument between the nucleocapsid and envelope. Cellular proteins such as actin, HSP70, Hsp90, β-tubulin, cofilin, and enolaseare components of the EBV tegument. Historically, the EBV genome was the first to be fully sequenced using a conventional sub-cloning and Sanger sequencing strategy. An isolate of the EBV type 1 strain (M 81), which shows an increased ability to infect the epithelial cells, a reduced ability to infect B-cells, and increased lytic replication, has been proposed as a new EBV subtype [13].

The typical features of EBV and most other Lymphocryptovirus (LCV) genomes include: [1] a single overall format and gene arrangement; [2] two to five tandem repeats, 0.5 kbp, direct repeats of the same sequence at both termini (TR); [3] six to twelve tandem reiterations of a 3 kbp, internal direct repeat (IR1); [4] short and long largely unique sequence domains (US and UL) that contain almost all of the genome coding capacity; [5] perfect and imperfect tandem DNA repeats, most of which are within ORFs; and [6] a duplicated genome region [13].

### EBV latency

During latent infection, EBV genomes persist as circular extrachromosomal elements (episomes) in the nuclei of infected cells. While random integration of the EBV genome into the host chromosome occurs in rare cases, it is not the primary mechanism of persistence. EBV gene expression during latency is highly restricted compared to the productive lytic cycle due to the episomal form. The episomes are complexed with host cell histones (e.g., H2A, H2B, H3, H4) and form a circular minichromosome structure, which is essential for the regulation of latent gene expression and long-term persistence in B-cells.

EBV employs a complex strategy of persistence, characterized by at least four distinct gene expression programs, referred to as latency types or programs (Latency 0, I, II, and III). Type 0 latency occurs in resting memory B-cells of healthy carriers, where the only viral genes expressed are the EBV-encoded small RNAs (EBERs) and BART microRNAs. In the restricted form of EBV latency, known as type I latency, the virus expresses only one viral protein, EBNA1, along with a few non-coding RNAs (EBERs and BART microRNAs). Based on the differential promoter activation, type II latency pattern can be further subdivided into latency IIa and IIb. The type IIa latency pattern is characteristic of EBV-associated malignancies such as nasopharyngeal carcinoma, Hodgkin lymphoma as well as subsets of EBV-associated T-cell and NK-cell lymphoproliferative disorders. In this type of latency, the virus expresses EBNA1, LMP1, LMP2A, LMP2B, EBERs, and BART miRNAs. In type IIb latency, LMP promoters are silenced and the virus expresses EBNA1, EBNA2, EBNA3A, EBNA3B, EBNA3C, EBNA-LP, EBERs, BHRF1, and BART miRNAs.

**Fig. 1:**
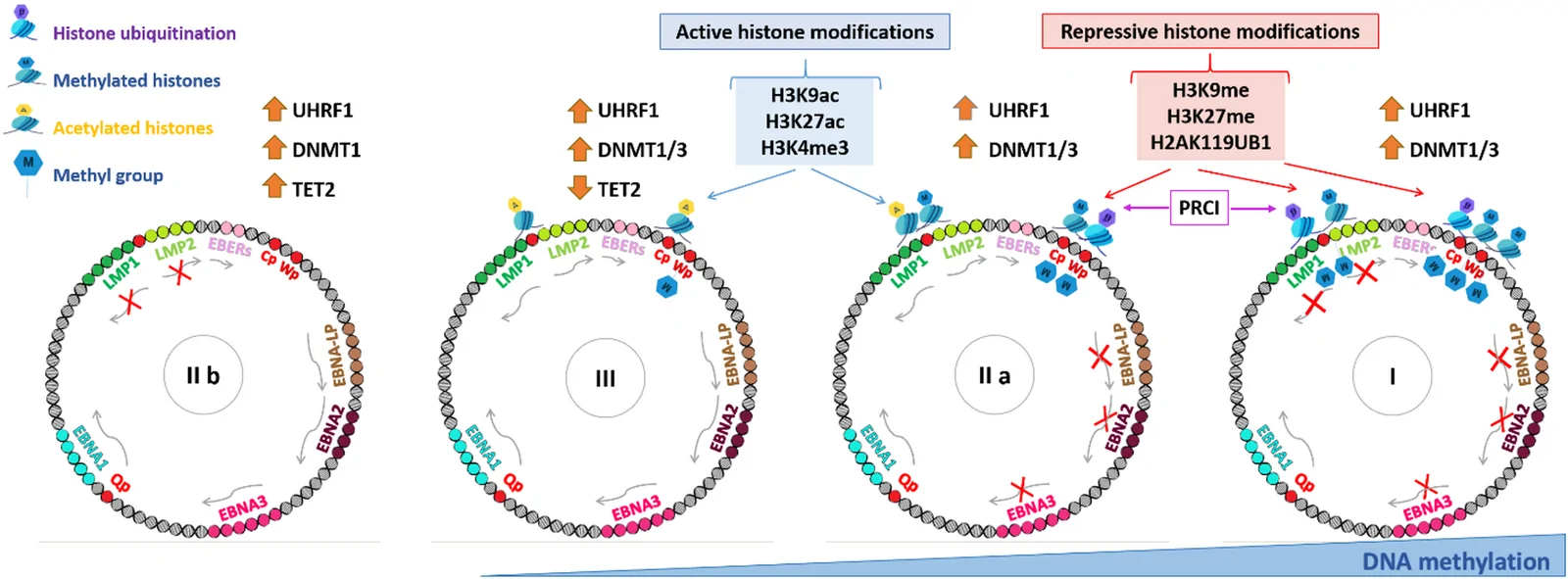
EBV epigenetic regulation and latency types. EBV utilizes DNA methylation and histone tail modifications to orchestrate the differential silencing and activation of viral promoters, thereby determining the phenotypic expression of latency types. DNA methyltransferases maintain the CpG methylation on the EBV episome to dictate the latency program. During the type IIb latency, the virus-induced upregulation of host DNA demethylase TET2, facilitates the active demethylation of viral promoters and enabling the robust transcription of the EBNAs. In restricted types of EBV latency (I and IIa), hypermethylation of the Cp and Wp promoters prevents the expression of most EBNA proteins. Active histone modifications generally indicate an open chromatin state that allows gene expression. In type III latency, the episome is highly enriched in histone active marks, particularly at the Cp and LMP1/2 promoters. Repressive histone modifications condense the viral genome to silence gene expression. Type I latency is characterized by high levels of repressive histone modifications at the silenced Cp, Wp and LMPs promoters

This state is often observed in primary infected B-cells. In lymphoblastoid cell lines (LCLs) and B-cell lymphomas, EBV expresses the full set of latency-associated genes (EBNA1, EBNA2, EBNA3A, EBNA3B, EBNA3C, EBNA-LP, LMP1, LMP2A, LMP2B, EBERs, BHRF1, and BART miRNAs), a pattern known as type III latency. In type I latency, the EBNA1 gene is transcribed from the Q or W promoter (Qp, Wp), while in type III latency, a polycistronic mRNA encoding for all the EBNA genes is initiated from the C promoter of the EBV genome [14, 15] (Fig. 1).

Host epigenetic states prior to EBV infection are critical in directing the virus toward specific latency types. Initial infection of naive B-cells often starts with type III latency, but the gradual accumulation of host-driven DNA methylation and repressive histone marks forces a transition to more restricted latency types (II and I). The epigenetic state induced by B-cell receptor (BCR) stimulation and cytokines within germinal centers allows for a shift from type III to type II latency. EBV exploits cell-type-specific epigenetic markers to establish a stable, silent episome while minimizing detection by the host immune system [16].

## Basic concepts of epigenetic mechanisms

In 1942, the British embryologist Conrad Waddington introduced the term epigenetics to describe the complex developmental processes and causal interactions between genes and their products [17]. Epigenetic change is a sophisticated regulatory mechanism that affects gene transcription without altering the DNA sequence itself. Epigenetic modifications are dynamic and flexible processes that can be altered or removed (reversible) and can be passed from parents to offspring, in certain cases, through the germline (heritable). Epigenetic modifications also play an important role in the silencing or expressing non-coding sequences (junk DNA) in the genome. Aberrant epigenetic patterns are a major factor in the occurrence and progression of a wide range of human diseases [18], such as viral infections, cancer, neurodegenerative disorders [19], metabolic disorders, cardiovascular disease, and autoimmune and inflammatory diseases.

The well-established types of epigenetic mechanisms are DNA methylation, histone modifications (such as acetylation, methylation, phosphorylation, and ubiquitination), and changes in non-coding RNA (ncRNA) biogenesis. DNA methylation is one of the most studied and prevalent epigenetic modifications in mammalian cells, typically occuring in the context of a CpG dinucleotide (promoters and enhancers). The process is catalyzed by a family of enzymes called DNA methyltransferases (DNMTs), which add methyl groups (CH3) to the fifth carbon of cytosine residues, resulting in the formation of 5-methylcytosine for regulating gene expression. EBV can use specific methylation patterns to maintain latency and promote tumorigenesis by silencing host tumor suppressor genes or activating oncogenes [20].

Histone modifications constitute a fundamental mechanism of epigenetic regulation that affects chromatin regulation and gene expression. These modifications are highly dynamic, and abnormal changes are strongly linked to the occurrence and progression of a wide range of diseases. EBV extensively utilizes and manipulates the histone epigenetic modifications of cells to regulate both its own life cycle (latency and lytic replication) and the host gene expression program [21]. Distinct patterns of histone modification have been observed in different types of EBV-associated disease.

Methylation of histone H3 at lysine 4 (H3K4) is generally associated with gene activation, whereas methylation at H3K9 and H3K27 is strongly linked to gene silencing by promoting a compact, inaccessible chromatin state [14]. Histone phosphorylation typically occurs on the serine residues of histone H3 and can either induce or inhibit gene expression, depending on the cellular context. Ubiquitination of histones H2A and H2B is a highly site-specific epigenetic modification and plays a critical role in both transcriptional activation and repression. For example, monoubiquitination of histone H2A at lysine 119 is commonly associated with gene silencing, while monoubiquitination of histone H2B at lysine 120 correlates with active gene expression [22]. Histone acetylation and deacetylation are crucial, reversible covalent modifications catalyzed by histone acetyltransferases (HATs) and histone deacetylases (HDACs), respectively. Acetylation typically occurs on lysine residues of histones H3 and H4 and has a chromatin-loosening effect, resulting in transcriptional activation. The dynamic and reversible nature of histone modifications makes them promising targets for the development of novel therapeutic interventions, particularly in the treatment of EBV-associated malignancies.

**Fig. 2:**
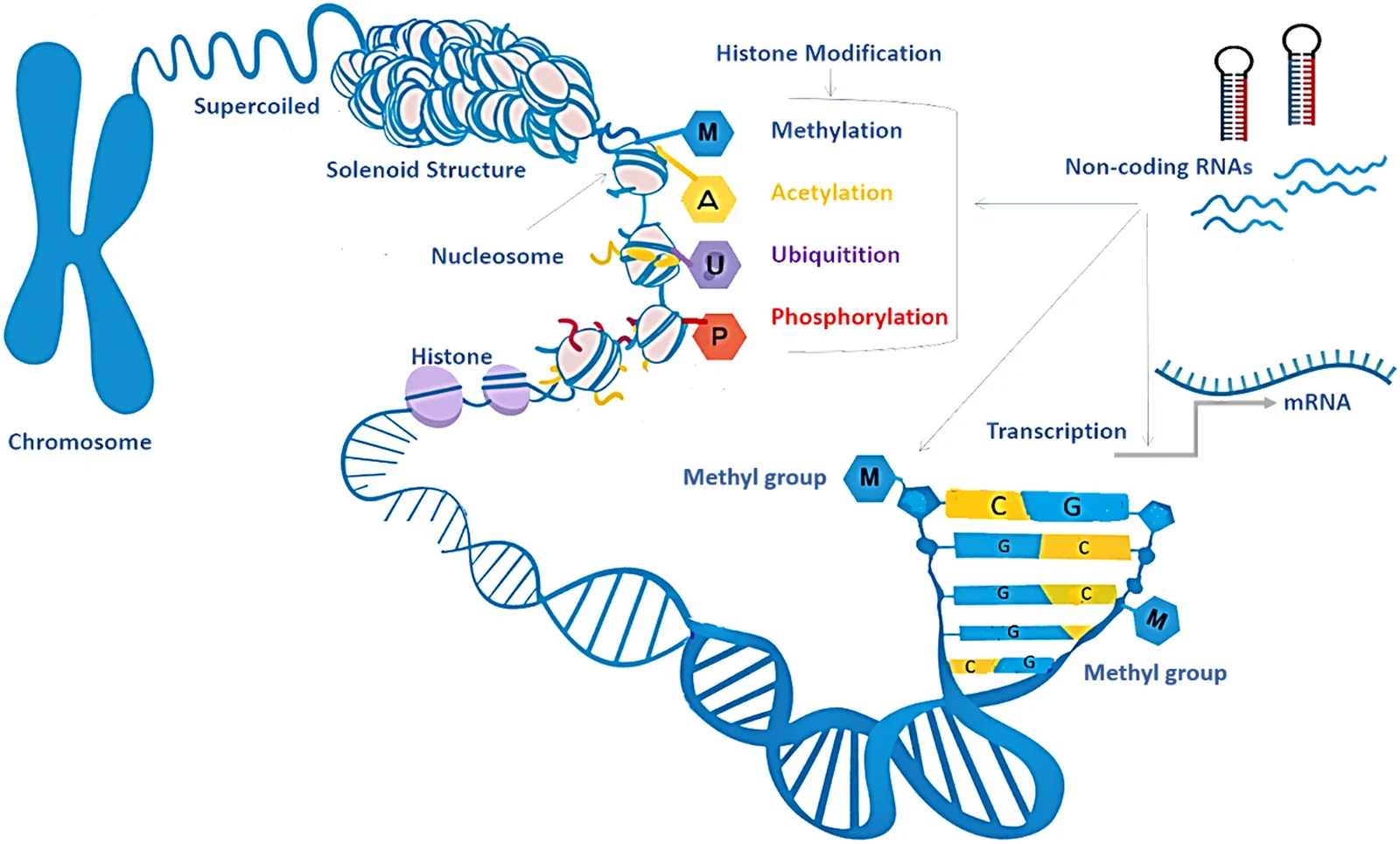
Overview of cellular epigenetic modifications. Chromosomal DNA forms nucleosomes and is packaged within the nuclei by histone proteins. Chromatin remodeling is regulated by various epigenetic modifications. Post-translational modifications (PTMs) of histone tails are crucial in controlling DNA accessibility within nucleosomes. Methylation of CpG sites in the promoter region of the gene can lead to gene silencing. Non-coding RNAs, such as miRNAs and lncRNAs, are regulatory molecules that play critical roles in controlling gene expression, DNA methylation, and histone modifications

Non-coding RNAs, such as long non-coding RNAs (lncRNAs) and microRNAs (miRNAs), play a vital and well-established role as key epigenetic regulators in both normal physiological processes and viral infections [23, 24]. During viral infection, both host- and virus-encoded ncRNAs are central to the dynamic interplay between the viruses and host immune responses. MiRNAs, as critical epigenetic regulators of gene expression, can directly target and regulate the expression of epigenetic enzymes such as DNMTs, HDACs, and polycomb group proteins, including polycomb repressive complex 1 and 2 (PRC), thereby influencing chromatin structure and DNA methylation patterns. In addition, miRNAs can localize to the nucleus and directly influence transcription by associating with specific gene promoters to either activate or repress gene expression. LncRNAs can recruit chromatin-modifying complexes to specific genomic locations and regulate gene expression [25]. These functions highlight the sophisticated role of ncRNAs as key coordinators of the cell’s epigenetic landscape (Fig. 2).

Exosomes are small extracellular vesicles (30–150 nm) released by many cell types that transport signaling molecules, such as ncRNAs, between cells, thereby inducing epigenetic changes in recipient cells [24]. The transfer of ncRNAs via exosomes can alter DNA methylation and histone modification patterns in cells[26]. Exosome-mediated transfer of information is vital for normal cellular function and the development of a wide range of diseases, including viral and bacterial infections, neurodegenerative disorders, autoimmune diseases, and inflammatory disorders [26].

## Epigenetic regulation in EBV infection

### Epigenetic alterations acquired on the EBV genome

EBV infection of epithelial cells and B-cells is significantly different in viral life cycle, epigenetic regulation, and pathogenesis. Normal nasopharyngeal epithelial cells are not permissive for long-term latent infection, and infection often leads to the lytic cycle with the production and shedding of new virions in saliva. Infection of B-cells establishes a lifelong latent infection, typically in memory B-cells, where the virus remains largely silent [27].

Linear EBV genomes are chromatinised and circularised early after infection, which is important for the virus to establish a lifelong latent infection within the host cell [28]. Epigenetic regulation is critical for EBV to control the switch between the lytic and latent phases. The lytic phase is triggered by a significant shift in EBV’s epigenetic state, producing unmethylated and unchromatinized DNA [29]. Multiple layers of epigenetic control are central to the EBV life cycle, enabling the virus to establish latency, control latency program selection, and ultimately support lytic reactivation [11].

The epigenetic alterations in the EBV genome within infected epithelial cells differ from those in B-cell infections [30]. While EBV regulates methylation levels to its own advantage in B-cells, its manipulation in the epithelial cells appears to promote carcinogenesis by globally disrupting the host’s epigenetic balance. EBV genome methylation levels progressively increase following B-cell infection, and this gradual methylation is crucial for long-term latency and the transition through different latency programs [31].

EBV infection in the oral cavity leads to extensive hypermethylation of the host cell genome, which is a key factor in the development of EBV-associated carcinomas, such as NPC and GC [14, 32]. Hypermethylation can silence tumor suppressor genes, cellular differentiation factors, and cell cycle regulators, resulting in a favorable environment for cancer development by promoting cell growth and proliferation. Moreover, hypermethylation can lead to genomic instability and long-lasting epigenetic changes in infected cells [33].

#### Histone modification

Histone modifications, including acetylation, methylation, phosphorylation, ubiquitination, and sumoylation, have various biological functions in viral infected cells. The chromatin composition of the EBV genome is highly dynamic, and the specific histone modifications such as methylation and acetylation present on the viral minichromosome correspond to the expected marks for either viral gene activation or repression. Upon EBV infection, Wp promoter is hypomethylated and enriched with active histone marks. As the virus moves into more restricted latency types, Wp is silenced by methylation and repressive histone marks.

In type III latency, the Cp and LMP1/LMP2 promoters are enriched with acetylated histones such as H3K9Ac, H3K4me3, and H3K27ac. These marks establish an open chromatin structure accessible to transcription factors and RNA polymerase, leading to transcriptional activation. In contrast, these marks are absent from Cp and LMP1/LMP2 in type I latency, where these promoters are inactive. In type I latency, activating marks that are responsible for EBNA1 expression are concentrated at the Qp promoter, while the remainder of the genome, including other latent and lytic gene promoters, is heavily enriched with repressive marks, including H3K9me3 and H3K27me3. Both latency types retain modest H3K27me3 enrichment at lytic immediate-early promoters, indicating that heterochromatin contributes to the maintenance of viral latency by restraining lytic reactivation [34, 35].

Chromatinization plays a key role in silencing EBV lytic genes in newly infected cells, with viral genomes becoming chromatinized over the first day post-infection. Histone chaperones, such as the DAXX-ATRX complex, are host cellular proteins that are important in controlling both cellular and viral gene expression, as well as in the formation and maintenance of heterochromatin. The DAXX-ATRX complex mediates the deposition of H3.3 and other repressive histone marks onto EBV genomes, rapidly compacting the viral genome into heterochromatin and silencing viral gene expression. The EBV major tegument protein, BNRF1, has been shown to bind to DAXX and disrupt the DAXX-ATRX complex, thereby activating viral gene transcription. This disruption is a critical step in overcoming the host’s first-line antiviral defense mechanisms and is essential for the induction of early viral gene expression, including EBNA2 [36].

PRC1 is an epigenetic regulator that primarily functions as a histone H2A ubiquitin ligase, adding a monoubiquitin mark at lysine 119 (H2AK119Ub1), which is generally associated with transcriptional repression. Studies using CRISPR/Cas9 screens in Burkitt’s lymphoma cell lines have shown that PRC1 is critical for silencing the LMP promoters (LMPp1 and LMPp2A) and may add a layer of epigenetic control over latency switching [37].

Recent studies have further refined the role of PRC1 in EBV latency control by identifying RING1A, a catalytic core component of canonical PRC1 complexes, as a key writer of the repressive H2AK119Ub mark at the BZLF1 (ZTA) promoter. Using locus-specific proteomics, chromatin immunoprecipitation, and CRISPR-Cas9 screening approaches, RING1A and multiple PRC1 components were shown to occupy the BZLF1 promoter and suppress lytic gene expression. Depletion of RING1A resulted in loss of H2AK119Ub, increased BZLF1 expression, and spontaneous lytic reactivation, indicating that PRC1-mediated H2AK119 ubiquitination is critical for maintaining EBV latency. Notably, PRC1 recruitment and repression of BZLF1 appeared to function largely independently of PRC2-mediated H3K27me3 deposition, revealing an additional layer of epigenetic regulation controlling the EBV latent–lytic switch [38].

PRC2 and H3K9 methyl transferases deposit repressive histone marks on the EBV genome, establishing and maintaining viral latency. The EBV lytic switch protein BZLF1 has been shown to counteract the repressive effects of H3K9me3 during reactivation [39].

Recent evidence has also identified H3K4 methylation as a critical regulator of the EBV latent–lytic switch. A genome-wide CRISPR-Cas9 screen in Bl cells demonstrated that lysine-specific demethylase 1 (LSD1/KDM1A), together with its cofactors CoREST and ZNF217, is essential for maintaining EBV latency. Mechanistically, the LSD1–CoREST–ZNF217 complex occupies key EBV regulatory regions, including oriLyt enhancers and the BZLF1 promoter, where LSD1 removes activating H3K4 methylation marks and restricts chromatin looping required for lytic reactivation. In opposition to LSD1 activity, the H3K4 methyltransferase KMT2D supports EBV lytic reactivation by promoting activating H3K4 methylation. These findings suggest that the balance between H3K4 methylation writers and erasers represents an important epigenetic switch controlling EBV latency and reactivation [40].

#### Methylation

EBV DNA methylation is a powerful mechanism by which the virus exerts precise control over both the selection of latency type and the suppression of lytic gene expression. EBV uses three main latency types (III, II, and I), each characterized by a distinct pattern of viral gene expression and a unique degree of DNA methylation, to navigate the B-cell compartment and establish long-term persistence in memory B-cells. As the B-cell differentiates and matures into a memory B-cell, methylation progressively increases at specific viral promoters. Methylation silences the Cp and LMP promoters, resulting in the highly restricted type I latency, in which only EBNA1 is expressed from the largely unmethylated Qp promoter. Moreover, Cp promoter methylation may contribute to the transition from type III latency to II [41]. An infectious EBV virion contains a linear double-stranded DNA genome that is unmethylated and not associated with cellular histones. The incoming genome remains largely unmethylated during the productive lytic cycle in epithelial cells. During the lytic cycle, the viral DNA undergoes extensive replication using viral enzymes. The newly synthesized EBV genomes also remain unmethylated and unchromatinized.

Epstein-Barr nuclear antigen 2 (EBNA2) is a transcriptional co-activator viral protein expressed during the latency phase. EBNA2 colocalizes with host cell transcription factors, such as EBF1 and RBP-Jκ, to regulate both viral and host cell transcription programs, without directly binding to DNA. The extent of viral genome methylation is limited by EBNA2, which induces the cellular cytosine demethylase TET2. In infected epithelial cells, the viral latent membrane protein 2 A (LMP2A) actively manipulates host epigenetic processes. LMP2A has been shown to upregulate the expression of DNA methyltransferases and downregulate demethylases (TET enzymes), which are the mechanisms underlying LMP2A-mediated hypermethylation.

Beyond EBNA1’s canonical role in viral episome maintenance, it binds to specific sites within the viral genome, such as oriP and the Qp promoter, and protects these regions from methylation, or even actively promotes local demethylation. EBNA1 binding interferes with the function of the host DNMT1 at these sites during viral replication, leading to passive demethylation. By contrast, EBNA1 can indirectly lead to the methylation and subsequent silencing of viral promoters, such as the C promoter, which helps transition the virus into more restricted latency types (I or II) with lower viral antigen expression, thus allowing EBV to evade host immune responses. In addition, methylation of EBNA1 affects its nucleolar localization, interactions with nucleolar proteins, and RNA binding [42].

DNMT3B is upregulated in normal human germinal center (GC) B-cells. In primary B-cell infection, both the epigenetic enzyme ubiquitin-like, containing PHD and RING finger domains 1 (UHRF1) and DNMT1 are upregulated in normal human GC B-cells compared to naive B-cells. As B-cells differentiate into memory B-cells and the EBV DNA becomes heavily methylated by DNMT3B at the germinal center stage, the virus enters the type I latency. DNMT1 and UHRF1 are responsible for maintaining DNA methylation marks on the EBV genome as B-cells differentiate into memory B-cells and keeping the viral lytic genes silenced. The highly restricted type I latency in Burkitt B-cells depends on DNA methylation to silence viral gene expression and the use of DNA hypomethylating agents, or depletion of UHRF1 leads to type III latency and lytic gene expression in infected cells [37, 43].

While DNA methylation generally acts as a repressive mark to silence the lytic cycle during latency, it also plays a critical role in efficient reactivation of the lytic cycle from latency. DNA methylation is required for the viral-encoded immediate early protein Zta (also referred to as ZEBRA, Z, and BZLF1) to bind the promoters of several essential viral genes. Most early lytic viral promoters are preferentially activated by BZLF1 in the methylated form. In fact, methylation of the viral genome enhances BZLF1 binding to CpG motifs in early lytic promoters. The Zta promoter (Zp) is a key control point for the switch from latency to the lytic cycle. The Zta protein, the master regulator of the lytic switch, can overcome the silencing of lytic genes by selectively binding to methylated DNA. Interestingly, the Zp promoter itself is primarily silenced during latency through several mechanisms distinct from DNA methylation, including sequence-specific repressor proteins, notably zinc finger E-box-binding homeobox 1 (ZEB1), repressive histone marks particularly H3K27me3, and higher-order chromatin structure [44, 45]. Thus, DNA methylation controls EBV gene expression programs in both negative (repressive) and positive (activating) ways during latency and the lytic cycle. BRLF1(Rta) is a viral-encoded immediate-early protein that acts as a transcription factor. Most early lytic viral promoters are preferentially activated by BZLF1 in the methylated form, while methylation decreases the ability of BRLF1 to activate most early lytic promoters. In addition, BRLF1 preferentially induces H3K9 histone acetylation of unmethylated promoters, facilitating their expression and leading to the expression of lytic cycle genes. These results suggest that the unmethylated form of the EBV genome can undergo viral reactivation and replication in a BRLF1-dependent manner [46]. This differential response to viral DNA methylation is an important regulatory switch that determines whether EBV establishes a latent infection in B-cells, where the genome becomes methylated or undergoes a productive lytic infection in epithelial cells, where the genome remains unmethylated.

Beyond histone modifications and DNA methylation, the higher-order DNA structure of the EBV genome within the host cell nucleus is essential for controlling latency type selection, suppressing lytic gene expression, and promoting viral persistence. The CCCTC-binding factor (CTCF) is a highly conserved, essential nuclear protein that acts as a multi-functional transcriptional regulator and chromatin insulator. CTCF is a critical organizer of the three-dimensional (3D) structure of the EBV genome. CTCF activity is regulated by DNA methylation and by the host enzyme poly (ADP-ribose) polymerase (PARP1). CTCF and PARP1 colocalize at specific EBV genomic sites, such as the Cp, Qp, and LMP1/2 promoters, to mediate long-range chromatin interactions or to insulate genome regions [47, 48].

In type I latency, CTCF forms a specific chromatin loop between OriP and the EBNA1 promoter (Qp). This chromatin loop structure ensures consistent expression of EBNA1 from Qp, while physically isolating other nearby gene promoters (such asCp and LMPp) within repressed heterochromatin domains. In type III latency, CTCF mediates looping between OriP and the C promoter, which is distal to Qp. This loop activates the Cp promoter, driving expression of all EBNAs, including the key transforming protein EBNA2. The change in looping conformation is driven by B-cell differentiation stage-specific transcription factors and DNA methylation patterns. These findings suggest that EBV promoter selection and latency switching are hierarchically organized processes regulated by epigenetic modulators, transcription factors, chromatin composition, and chromatin folding [47, 49].

#### Non-coding RNAs

Non-coding RNAs play crucial and diverse regulatory roles in EBV pathogenesis. EBV expresses various ncRNAs, including EBV-encoded RNAs (EBERs), the BamHI-A rightward transcripts a small nucleolar RNA (snoRNA), and viral microRNAs (miRNAs). EBV-encoded non-coding RNAs such as EBERs and viral microRNAs promote immune evasion, sustain latent infection, and regulate viral and host transcriptional networks. Moreover, viral non-coding RNAs contribute to viral persistence, cellular transformation, and the development of EBV-associated malignancies. EBERs, including EBER1 and EBER2,are two small, non-polyadenylated, and the most abundant non-coding RNAs transcribed by host RNA polymerase III in infected cells. EBER1 and EBER2 are short, 167, and 172 nucleotides in length respectively, with 54% sequence homology. These non-coding RNAs are located in the BamHI C fragment of the EBV genome, separated by about 160 base pairs [50].

EBERs have a highly conserved structure across different EBV isolates and are mainly localized in the nucleus but can also be found in the cytoplasm of EBV-infected cells. The conserved structure is primarily formed through intramolecular base pairing, resulting in several stem-loops or hairpin loops. These complex secondary structures are essential for their function, as they enable the EBERs to interact with various proteins. Studies have shown that EBV-infected cells manipulate the local microenvironment by releasing exosomes containing both viral and cellular components, including EBERs. The secretion of EBERs via exosomes is an important viral strategy to alter the surrounding environment, evade immune surveillance, and promote the development and progression of EBV-associated malignancies [51].

EBV generates 44 different mature miRNAs, categorized into BamHI fragment H rightward open reading frame 1 (BHRF1) miRNAs and BamHI-A rightward transcript (BART) miRNAs, which directly or indirectly regulate EBV replication. BHRF1-1 overexpression in NPC cell line increases the EBV copy number, followed by the accumulation of the BZLF1 and BMRF1 lytic proteins [52]. Several EBV-encoded miRNAs, especially members of the BART and BHRF1 clusters, target the viral immediate-early transcription factors BZLF1 (Zta) and BRLF1 (Rta) at the mRNA level. These miRNAs downregulate the expression of viral immediate-early transcription factors and suppress lytic DNA replication, resulting in long-term episomal maintenance. The BHRF1 miRNAsare highly expressed following infection of primary B-cells and also after lytic reactivation, indicating potential functions in the transition between lytic and latent states [53].

BHLF1 encodes a 2.5 kb unspliced, polyadenylated RNA (a viral long non-coding RNA) that has distinct but related functions during both the lytic cycle and latent phase of EBV infection. Transcription of BHLF1 and synthesis of the GC-rich BHLF1 RNA promote the formation of stable RNA–DNA hybrids (R-loops) at oriLyt, which facilitate the recruitment and assembly of viral and host factors, such as the single-stranded DNA binding protein BALF2, thereby enabling initiation of lytic DNA replication. BHLF1 is highly expressed upon induction of the lytic cycle in latently infected B-cell lines. BHLF1 may also act as a structural or scaffolding component in virus-induced nodular structures (VINORCs), which are involved in pre-mRNA processing and nuclear export of viral mRNAs during the lytic phase. Recent evidence indicates that BHLF1 transcripts are consistently detected in types I and III latency, where its lncRNA contributes to key aspects of latency and B-cell immortalization. Studies in cell culture have shown that EBV lacking the BHLF1 locus is unable to sustain full type III latency and transitions more rapidly to the highly restricted type I latency, suggesting that BHLF1 is required to maintain the active type III latency state crucial for initial B-cell growth and immortalization. BHLF1 functions in latency as a lncRNA, possibly involving regulation of splicing of EBNA mRNAs or other post-transcriptional mechanisms [54, 55].

Recent studies have expanded the role of viral non-coding RNAs in R-loop biology beyond BHLF1. Wang et al. identified a novel EBV-derived stable intronic sequence RNA (ebv-sisRNA-3), generated from the BART region, which accumulates in latently infected epithelial cells and promotes the formation of R-loops upstream of OriLyt R. These R-loops are associated with G-quadruplex structures within the IR4 region and may contribute to regulation of lytic replication initiation. In contrast to the previously described BHLF1-associated R-loops that are primarily linked to early lytic replication, EBV-sisRNA-3-mediated R-loops are present during latency, suggesting that R-loop-dependent regulation participates in both latent and lytic phases of the EBV life cycle [56].

The balance between the control mechanisms mediated by EBV non-coding RNAs is a critical factor determining the efficiency, timing, and cell-type specificity of EBV genome replication, which enables EBV to establish a persistent infection while minimizing host immune detection.

#### RNA methylation

RNA methylation has recently emerged as an important epigenetic mechanism regulating multiple stages of the EBV life cycle, including latency establishment, maintenance of viral persistence, lytic reactivation, immune evasion, and host antiviral responses, represents a rapidly expanding area of EBV epigenetic research. Among the more than 100 known RNA modifications, N6-methyladenosine (m6A) is the most extensively studied in EBV biology. m6A modification is deposited by methyltransferase complexes containing METTL3 and METTL14 (“writers”), removed by demethylases such as ALKBH5 and FTO (“erasers”), and recognized by m6A-binding proteins including members of the YTH domain family (“readers”). Accumulating evidence indicates that m6A modification influences the stability, translation, and turnover of both viral and host transcripts, thereby affecting multiple stages of the EBV life cycle.

Recent studies have identified the m6A reader YTHDF2 as an important antiviral restriction factor during EBV infection. YTHDF2 promotes degradation of m6A-modified viral transcripts and limits EBV lytic replication [57]. During EBV reactivation, caspase-mediated cleavage of YTHDF2 and other components of the m6A machinery attenuates this antiviral pathway, thereby facilitating viral replication. Furthermore, PIAS1-mediated SUMOylation enhances the antiviral activity of YTHDF2 by increasing its ability to recognize and destabilize EBV transcripts. These findings demonstrate that EBV actively manipulates the host m6A machinery to promote efficient lytic replication [58].

RNA methylation also contributes to regulation of innate immune responses during EBV infection. Downregulation of the m6A demethylase ALKBH5 during lytic reactivation alters methylation-dependent regulation of DTX4 and TYK2, leading to suppression of interferon signaling and facilitating productive viral replication [59]. These observations reveal an important link between epitranscriptomic regulation and immune evasion during EBV infection.

Beyond m6A, emerging evidence suggests that 5-methylcytosine (m5C) modification also participates in gammaherpesvirus biology. Recent studies demonstrated that EBV suppresses the m5C methyltransferases NSUN1 and NSUN2 through c-Myc-dependent mechanisms, resulting in reduced m5C modification and destabilization of antiviral factors such as TRIM25. Consequently, RIG-I-mediated antiviral signaling is weakened, promoting viral replication and viral persistence. Together, these findings indicate that multiple RNA methylation pathways cooperate with other epigenetic mechanisms to regulate EBV latency, reactivation, immune evasion, and pathogenesis[60].

## EBV manipulation of the host epigenetic machinery

EBV encodes several viral factors that interact with host epigenetics and actively manipulate both viral and host epigenetic mechanisms (Table 1). EBV manipulates epigenetic machinery to transition between different latency programs, each characterized by specific gene expression patterns and associated diseases (Table 2). The primary function of EBNA1 in EBV latent infection is as a transcriptional regulator and transactivator of cellular promoters. EBNA1 is an essential, multifunctional EBV protein that binds the latent origin of replication (oriP) in its unmethylated state, and induces DNA looping, bringing EBNA1 into proximity with the Cp and LMP1 promoters to facilitate their activation. EBNA1 manipulates the epigenetic landscape of immune cells, primarily B-cells, and this manipulation plays a key role in EBV persistence, immune evasion, alteration of host chromatin structure, and the development of autoimmune diseases.

**Table 1:** EBV-encoded genes with therapeutic potential affecting host cell epigenetic mechanisms

Viral gene	Cellular epigenetic effects	Therapeutic potential
EBNA1	Alter chromatin architecture Alter methylation machinery Activate histone marks	Inhibitors of EBNA1 DNA-binding can disrupt viral persistence
EBNA2	Interact with HATs and chromatin remodelers Regulate host microRNAs Regulate demethylase	Target for blocking B-cell transformation
EBNA3C	Interact with HDACs and HATs Alter methylation machinery Recruit components of the PRC2 complex and deposition of the repressive histone mark	Target for reversing silencing of tumor suppressors in B-cell lymphomas
LMP1	Induce the formation of a transcriptional repression complex comprising DNMT1 and HDAC Alter methylation machinery	Targeting can reduce promoter hypermethylation of tumor suppressor genes
LMP2	Upregulate the expression of methyltransferases Downregulate the expression of demethylases	Targeting can reverse the specific host-cell silencing and oncogenic reprogramming
Viral non-coding RNAs	Recruit chromatin regulators Regulate host gene expression through histone methylation and acetylation Modulate miRNA activity Manipulate the local microenvironment via exosomes	Target for reducing tumor growth

EBNA, Epstein-Barr virus nuclear antigen; LMP, latent membrane protein; HATs, histone acetyltransferases; HDACs, histone deacetylases; DNMT, DNA methyltransferase; PRC, polycomb repressive complex

**Table 2:** EBV latency types and disease-specific epigenetic alterations

Latency types	Gene expression patterns	Key epigenetic marker	Diseases
0	EBERs, BART microRNAs	Cp/Wp/ Qp (Silenced/Methylated) Repressive histone modifications	Healthy carriers
I	EBERs, EBNA1, BART microRNAs	Qp (Active) Cp/Wp (Silenced/Methylated) Repressive histone modifications	Burkitt lymphoma, Gastric carcinoma
IIa	EBERs, EBNA1, LMPs, BART miRNAs	LMPs/ Qp (Active) Cp/Wp (Silenced/Methylated) Repressive histone modifications	Hodgkin lymphoma, NK/T-cell lymphoma, Nasopharyngeal carcinoma
IIb	EBERs, EBNA1, EBNA2, EBNA3A/B/C/LP, BHRF1, BART miRNAs	Cp/Wp (Active) Hypomethylation	Acute infectious mononucleosis, HIV-associated lymphoma, Post-transplant lymphoma, Diffuse large B-cell lymphoma
III	EBERs, EBNA1, EBNA2, EBNA3A/B/C/LP, LMPs, BHRF1, BART miRNAs	Cp/Wp (Active) Active histone modifications	Post-transplant lymphoproliferative disorder, Infectious mononucleosis, HIV-associated lymphoma, Diffuse large B-cell lymphoma

EBNA1 can bind to multiple sites across the host genome, particularly CpG-rich promoters and enhancer elements, and modulates the expression of immune-related genes primarily through direct DNA binding and by influencing local chromatin architecture. Studies have shown that EBNA1 influences the host DNA methylation machinery, thereby reshaping both viral and cellular epigenetic landscapes. EBNA1 can induce the expression of host DNA methyltransferases, specifically DNMT3a, through the E2F1 transcription factor. Increased levels of methyltransferases lead to broader methylation changes in the host DNA, and DNMT3a knockdown prevents cell proliferation, promotes cell apoptosis, induces cell cycle arrest, and suppresses cell migration [61].

EBNA1 interacts with various host epigenetic regulatory factors, including protein arginine methyltransferases (PRMT1 and PRMT5), to mediate changes in both viral and host gene expression. PRMT1 and PRMT5 have a variety of cellular and viral targets, thereby affecting diverse processes, including gene expression, signal transduction, transcription regulation, RNA splicing, and DNA repair. Studies show that the RGG motifs within the EBNA1 N-terminal domain are crucial for RNA binding. Methylation of EBNA1 is associated with RNA binding, and the Gly-Arg-rich region of EBNA1 is modified by both serine phosphorylation and arginine methylation [62, 63]. Through these mechanisms, EBNA1 can cause aberrant methylation patterns in the host genome, leading to dysregulating the host cellular gene expression, silencing tumor suppressor genes, and activating oncogenes such as MUC1-C [64]. The CCCTC-binding factor (CTCF) is a multifunctional protein that organizes chromatin architecture and plays several roles in the regulation of gene expression. The functional similarities between CTCF, EBNA1, and FoxA transcription factor suggest that EBNA1 may be capable of epigenetic reprogramming in EBV-infected cells, which could result in tumorigenesis [65]. EBNA-1 enhances the expression of the host cell transcription factor including activating transcription factor 3 (ATF3), by binding to an enhancer region upstream of the ATF3 gene and ATF3 promotes cellular gene expression with activating histone marks such as H3K27ac. Dysregulation of the host epigenetic landscape promotes the expression of target genes involved in cancer development, ultimately contributing to genomic instability and malignant transformation [66].

EBNA2 lacks a DNA-binding domain and is recruited to specific sites in the host genome by cellular DNA-binding proteins such as RBP-Jκ and PU.1. EBNA2 contributes to immune modulation by acting as a transcriptional activator, recruiting HATs and chromatin remodelers, which leads to an open chromatin state and gene expression. EBNA2 plays a key role in immune evasion after infection by suppressing genes associated with immune responses. EBNA2 induces the expression of the immune checkpoint molecule PD-L1, helping the infected cell evade T cell responses [67]. Studies have shown that EBNA2 can render infected cells less sensitive to interferons. It also regulates certain host microRNAs (such as miR-21, miR-146a) that act as negative feedback regulators of type I IFN responses.

EBNA2 interacts with HATs compartments, such as p300, CBP, and PCAF, as well as chromatin remodeling complexes, to remodel both the viral and host cell epigenomes, resulting in an open chromatin state and increased gene expression. The C-terminal domain (C-TAD) of EBNA2 recruits HATs to specific chromatin regions. This recruitment increases histone H3 and H4 acetylation, particularly H3K27 acetylation, at viral and host gene promoters and enhancers, thereby promoting gene expression. EBNA2 utilizesliquid-liquid phase separation (LLPS) mechanism to form nuclear condensates that facilitate the formation of accessible chromatin domains (ACDs), where p300 is recruited to promote H3K27 acetylation for epigenetic regulation [68]. EBNA2 also interacts with chromatin remodeling factors, such as the SWI/SNF complex member BRG1 ATPase, to orchestrate changes in chromatin structure and create an environment that supports viral persistence and oncogenesis. EBNA2 acts as an adaptor protein, recruiting chromatin remodeling factors to specific regulatory regions of both viral and cellular genes, such as the CD21, CD23, TP1, and LMP2A promoters, thereby enhancing gene expression, B-cell growth transformation, and immortalization [69].

EBNA2 has been shown to coordinate the regulation and function of Ten-Eleven Translocation 2 (TET2) DNA demethylase, a cellular tumor suppressor. The TET2 gene contains EBNA2-dependent binding sites and interacts with EBNA2 to demethylatethe genes important for EBV-driven B-cell growth transformation and maintenance of type III latency gene expression. Studies have shown that expression of TET1, TET2, and TET3 can be downregulated in various cancers. TET2 downregulation correlates with globally decreased 5-hydroxymethylcytosine (5hmC) and increased 5-methylcytosine (5mC), but EBNA2 and TET2 function cooperatively to maintain a relatively demethylated state that is permissive for viral gene expression [70, 71]. By forming new chromosomal binding sites and recruiting host transcription factors such as EBF1 and RBP-jκ to specific sites, EBNA2 leads to hypomethylation and activation of enhancer regions controlling critical B-cell growth and survival genes, including MYC, BCL2, and EBF [72]. EBNA2 also interacts with the human SNF5/INI1 protein (hSNF5/Ini1), a component of the hSWI/SNF chromatin remodeling complex, to facilitate nucleosome remodeling at its target genes for transcription. This mechanism is crucial for the growth-promoting and immortalizing effects of EBNA2 in infected cells [69].

EBNA3C can regulate viral and cellular epigenetic events that require acetylation or deacetylation by recruiting host factors to the promoters of specific viral and cellular genes, acting as either a transcriptional activator or repressor. EBNA3C directly interacts with HDAC1 and HDAC2 that are mediated mostly by cellular corepressors such as mSin3A, NCoR, and the DNA-binding protein RBP-Jκ [73]. EBNA3C promotes histone deacetylation at target promoters by recruiting HDACs, leading to condensed chromatin and transcriptional repression of tumor suppressor genes such as p16INK4a, p15INK4b, and Bim (BCL2L11). In addition, EBNA3C together with EBNA3A recruits the components of the PRC2 complex, including the methyltransferase EZH2, to specific gene promoters, resulting in the deposition of the repressive H3K27me3 histone mark, which is essential for silencing key cell cycle regulators and apoptosis-related genes [74, 75]. These cellular epigenetic regulations support virus-induced oncogenesis, contribute to B-cell immortalization, and facilitate immune evasion.

EBNA3C physically associates with the HATs p300 and CBP. Although these interactions suggest a role in acetylation and gene activation, EBNA3C has been shown to downregulate p300/CBP-mediated acetylation activity, particularly when complexed with the nuclear protein prothymosin alpha (ProTα). This modulation helps balance cellular transcription events and could interfere with the activity of other viral proteins, such as EBNA2, which typically recruits p300/CBP and chromatin remodeling factors for activation [76].

The Ras association domain family isoform 1 A (RASSF1A) is a cellular tumor suppressor that regulates a broad range of cellular functions, including cellular homeostasis, differentiation, cell cycle arrest, mitotic arrest, and apoptosis. Downregulation of RASSF1A expression by epigenetic silencing is one of the most common events in human cancers. Hypermethylation of the RASSF1A promoter silences the expression of mRNA. Moreover, treatment with a DNA methylation inhibitor, such as 5-Aza-2’-deoxycytidine, or DAC could restore RASSF1A expression. EBNA3C can decrease the transcriptional activity of the RASSF1A promoter by enhancing its methylation through indirect modulation of DNMT expression, resulting in B-cell proliferation and transformation [77, 78]. A comprehensive understanding of EBNAs-mediated epigenetic regulation of cellular functions facilitates the development of novel therapeutic approaches to target EBV-associated disorders.

LMP1 induces the formation of a transcriptional repression complex comprising DNMT1 and HDAC, leading to hypermethylation and silencing of various host tumor suppressor genes, such as the E-cadherin gene. LMP1 activates the c-Jun N-terminal kinase (JNK) signaling pathway through its C-terminal domain, resulting in the activation and binding of the AP-1 transcription factor complex to the DNMT1 promoter. AP-1 binding enhances the transcription and expression of the DNMT1 gene and increases the overall level of DNA methyltransferase in the cell. Recruitment of the DNMT1-HDAC complex to the promoter region of the E-cadherin gene causes local hypermethylation and histone deacetylation, resulting in a closed chromatin state and subsequent silencing of E-cadherin expression. Downregulation of E-cadherin promotes epithelial-mesenchymal transition (EMT) and enhances the development and invasive ability of carcinoma cells, predominantly in NPC [79, 80].These epigenetic alterations orchestrated by LMP1 can promote B-cell immortalization and provide a useful model for studying EBV-associated epigenetic changes contributing to malignancy.

Studies in EBV-associated GC and B-cells have shown that LMP2A activates DNMT1 through phosphorylation of the signal transducer and activator of transcription 3 (STAT3) pathway. Activated STAT3 translocates to the nucleus and acts as a transcription factor, leading to increased transcription and subsequent overexpression of the DNMT1 gene, which subsequently results in the hypermethylation and stable silencing of host tumor suppressor genes, such as PTEN (phosphatase and tensin homolog), thereby contributing to the oncogenic process. In addition, LMP2A causes upregulation and overexpression of the cellular transcription factor, EHF, through activation of STAT3. EHF binds to and activates the promoter region of oncogenes, such as Frizzled-5 (FZD5), which are involved in cell proliferation and survival in EBV-infected cells [81, 82]. Therefore, these findings highlight LMP2A as a critical host epigenetic modulator that subverts normal host signaling pathways to establish an environment conducive to viral persistence and oncogenesis.

EBV encodes a diverse collection of non-coding RNAs to regulate, often indirectly, host epigenetic mechanisms, primarily by manipulating host signaling pathways, post-transcriptionally repressing host genes, and inhibiting immune responses. Studies have shown that most viral non-coding RNAs are implicated as epigenetic regulators by recruiting transcription factors and chromatin regulators to target sites or by modulating the expression of chromatin remodelers. EBER2, an abundant viral non-coding RNA, was shown to target the cellular transcription factor PAX5 to the viral genome, suggesting that EBV non-coding RNAs may guide the recruitment of transcription factors not only to the viral promoter but also to the cellular promoter [83, 84].

BART lncRNAs play a critical role in modulating the host cell epigenetic mechanisms, which facilitate EBV latency, immune evasion, cancer progression, and metastasis. BART lncRNA is associated with the CREB-binding protein (CBP/p300) complex in the cell nucleus, resulting in the regulation of host gene expression, which mediates this through histone methylation and acetylation. BART lncRNA appears to regulate cellular RNA polymerase II (Pol II) function at the promoter region of certain genes as knockdown of BART lncRNA in NPC cells leads to an increase in the expression of interferon genes, cytokine genes, interferon-stimulated genes (ISGs), and chemokine-related genes, indicating that BART lncRNA may modulate the host immune response to facilitate immune evasion [85]. In addition, BART lncRNAs orchestrate extensive cell epigenetic and post-transcriptional reprogramming by modulating miRNA activity. BART expression reprograms the cellular miRNA and lncRNA regulatory network, thereby enhancing proliferation, survival, invasion, and transformation during tumor development, growth, and progression [86].

Studies have shown that the NF-κB signaling pathway activates the BART promoter, and high NF-κB activity in EBV-infected epithelial cells correlates with higher expression levels of both BART miRNAs and lncRNAs. Moreover, LMP1 is a potent activator of the NF-κB pathway, thereby leading to the upregulation of BARTs expression. Interestingly, some BART miRNAs, such as miR-BART5-5p, can downregulate LMP1 expression, creating a negative feedback loop that carefully balances the production of viral proteins to avoid triggering a strong host immune response while maintaining the persistent latent infection and oncogenic process [87, 88]. p53 up-regulated modulator of apoptosis (PUMA) is a pro-apoptotic protein belonging to the Bcl-2 family that is essential for p53-mediated apoptosis. PUMA and p53 are the main regulators of cellular growth and apoptosis, and the regulation of PUMA by miR-BART5, which is abundantly expressed in NPC and GC, leads to the establishment of latent infection and host cell survival [89].

Host cell ncRNAs play a crucial and dynamic role during EBV infection. The virus significantly alters host cell ncRNAs expression, and this dysregulation is a key component to evade the immune system, establishing lifelong latency and promoting cellular transformation. EBV infection leads to a significant increase or decrease in the host ncRNAs profile, particularly in long lncRNAs and miRNAs. In addition, EBV miRNAs target human mRNAs involved in transcription, apoptosis, Wntsignalling, and cell cycle control [90].

Metastasis-associated lung adenocarcinoma transcript 1(MALAT1) is an infrequently spliced lncRNA, which is highly expressed in the nucleus and involved in regulating gene expression at both transcriptional and post-transcriptional levels. MALAT1 could act as an oncogene to promote tumorigenesis and contribute to immune evasion, as reported to be upregulated in certain lymphoid malignancies, including DLBCL and some T-cell lymphoproliferative disorders. MALAT1 contributes to the ability of tumor cells to evade the immune system by functioning as a molecular sponge that sequesters the cellular tumor-suppressive microRNA (miR-195), which in turn leads to the upregulation of the immune checkpoint protein PD-L1 on the surface of the tumor cells. PD-L1 interacts with the PD-1 receptor on the surface of T-cells, transmitting an inhibitory signal that suppresses T-cells activation and proliferation [91, 92]. Moreover, several host lncRNAs are found to be significantly increased or decreased in EBV-infected cells and associated cancers such as LINC00312, NORAD, CYTOR, and SNHG8; their regulation by EBV contributes to the maintenance of viral latency, evasion of host immune surveillance, promotion of cell proliferation, and tumorigenesis [88, 92].

EBV also regulates the expression of nuerous cellular miRNAs, such as miR-155, miR-146a, miR-21, miR-29, and miR-34a, which influence cellular processes and immune responses. The upregulation of miR-21 drives B-cells into a proliferative/transformation state, and studies have shown that EBNA2 is a key viral protein responsible for increasing miR-21 levels in infected B-cells. In addition, the low levels of miR-146a found in EBNA2-expressing cells correlate with the ability of EBV to evade innate antiviral responses. The reduced levels of miR-146a in infected cells are associated with an increased expression of its target, IRAK1, and an altered type I interferon response [93]. MiR-155 is the most frequently upregulated miRNA in EBV-related B-cell malignancies. EBNA2 can upregulate miR-155 expression through both direct and indirect mechanisms, and LMP1 activates miR-155 transcription through the NF-κB and AP-1 pathways. EBNA2 directly activates the transcription of the miR-155 gene (miR-155HG or BIC) by targeting a specific enhancer element located upstream of the transcription start site. EBNA2 can also indirectly upregulate miR-155 expression by increasing the expression of the host transcription factor IRF4 (interferon regulatory factor 4) [94, 95]. Therefore, understanding the interplay between EBV and host non-coding RNAs is crucial for identifying potential therapeutic targets for EBV-related diseases.

## Therapeutic potential of epigenetic manipulations

Epigenetic-based therapies are an emerging and promising approach for treating chronic and oncogenic viral infections such as EBV infection.Accumulating evidence indicates that alterations in host epigenetics occur during EBV infection, and viral gene expression is controlled by epigenetic mechanisms. Furthermore, epigenetic modifications play a crucial role in regulating every stage of the EBV life cycle, from initial infection and the establishment of lifelong latency to the transition into the lytic phase and the promotion of oncogenesis. Epigenetic drugs modulate host enzymes such as DNMTs, HDACs, and HMTs and their associated cellular processes, which the virus hijacks for its own replication, latency, and immune evasion.

This offers the possibility of developing broad-spectrum antivirals that are less prone to viral resistance than conventional antiviral drugs. Additionally, the reversible nature of epigenetic modifications makes them attractive drug targets for novel therapeutic interventions.

Over the past two decades, there has been a significant growthin the investigation and use of small-molecule drugs targeting epigenetic mechanisms, particularly in oncology. Epigenetic therapy and epigenome editing have transformed cancer treatment, and numerous epigenetics-targeted drugs are undergoing clinical trials. Furthermore, advances in epigenetic analysis and molecular modification techniques, such as high-throughput sequencing technologies, proteolysis targeting chimera(PROTAC), nanomedicine, drug delivery systems, and CRISPR/Cas, facilitate the identification of specific epigenetic biomarkers for early diagnosisand have accelerated the clinical adoption of these drugs.Currently, epigenetic drugs that have been approved for marketing are categorized into different types based on their mechanisms [96].

Histone acetylation-targeted drugs, particularly histone deacetylase (HDAC) inhibitors, are actively being investigated for the treatment of EBV-associated malignancies. HDAC inhibitors promote histone hyperacetylation at viral promoters and can induce partial or complete reactivation of the EBV lytic cycle in certain cellular contexts. This property has led to the development of “lytic induction therapy” strategies, in which expression of lytic viral kinases may enhance the activation of antiviral prodrugs such as ganciclovir and increase the immunogenicity of EBV-positive tumor cells. However, the therapeutic consequences of EBV lytic reactivation remain context-dependent and incompletely understood. While lytic induction may increase susceptibility of tumor cells to antiviral agents and immune-mediated clearance, EBV reactivation has also been associated with inflammatory responses and immune dysregulation. Therefore, current therapeutic approaches generally aim to combine lytic induction with antiviral or immunotherapeutic strategies rather than relying on lytic reactivation alone. HDAC inhibitors, including belinostat, vorinostat, nanatinostat, and romidepsin, remain among the most extensively studied epigenetic agents in EBV-associated cancers.

Romidepsin is an FDA-approved HDAC inhibitor for treating cutaneous T-cell lymphoma (CTCL) and peripheral T-cell lymphoma (PTCL). Vorinostat is FDA-approved for the treatment of CTCL. Vorinostat has shown activity in PTCLs during clinical trials but does not currently hold a specific FDA approval for PTCL. Vorinostat and romidepsin have demonstrated the ability to upregulate BZLF1 and BRLF1, key transcriptional activators of the EBV lytic switch. Induction of lytic gene expression, they might increase the susceptibility of EBV-positive tumor cells to antiviral agents and immune-mediated elimination, supporting ongoing investigation of HDAC inhibitors as components of combination therapeutic strategies for EBV-associated malignancies [97, 98]. Belinostat, which is FDA-approved for the treatment of PTCL, has been shown in preclinical studies to have similar epigenetic reactivation effects in EBV-positive B-cell lines and to enhance immunogenicity [99]. Belinostat targets a broad range of HDAC enzymes (pan-HDAC inhibitor) but its use in EBV-positive cancers is largely investigational and focuses on its ability to disrupt viral latency. Nanatinostat is a class-I selective oral histone deacetylase inhibitor that induces the expression of lytic EBV genes. Nanatinostat and valganciclovir combination has received FDA fast track designation for EBV-positive lymphomas and clinical trials continue to evaluate the efficacy and safety of this combination for EBV-associated malignancies [100]. Chidamide, an orally administered selective HDAC inhibitor, is currently under investigation for a variety of solid and hematological malignancies [101]. Clinical research (Phase 2 Trial) has shown that chidamide has promising activity specifically in EBV-associated lymphomas. In China, chidamide is approved for treatment of EBV-driven cancers [102].

Histone demethylase-targeted therapy has recently emerged as an additional strategy for EBV lytic induction. LSD1/KDM1A acts as an H3K4 demethylase that maintains EBV latency by removing activating H3K4 methylation marks from viral regulatory elements. Pharmacological inhibition of LSD1 using selective inhibitors such as SP-2509/C12 induces EBV lytic reactivation in EBV-positive lymphoid and epithelial tumor models. Moreover, dual LSD1/HDAC inhibitors such as corin might enhance lytic induction by simultaneously preventing the removal of activating H3K4 methylation marks and increasing histone acetylation. Importantly, LSD1 inhibition sensitizes EBV-positive tumor cells to ganciclovir-mediated cytotoxicity in preclinical xenograft models. These findings highlight the role of LSD1 as a promising therapeutic target and support the development of combination epigenetic strategies that pair lytic induction with antiviral therapy [40].

Histone methyltransferases (HMTs) modify and maintain cellular epigenetic signals that influence chromatin structure to regulate gene transcription. Inhibitors of HMTs are extensively investigated as potential therapeutic agents in various malignancies. Enhancer of zeste homolog 2 (EZH2), a catalytic subunit of PRC2, which deposits the repressive H3K27me3 histone mark, is deregulated in various types of cancer. Tazemetostat, the first oral EZH2 inhibitor, received FDA approval in 2020 for select sarcomas and lymphomas epigenetic therapy. The recommended dose of tazemetostat is 800 mg orally, twice daily, until disease progression or unacceptable toxicity [103]. In EBV-associated cancers, viral proteins recruit the PRC2 complex to silence host tumor suppressor and pro-apoptotic genes, such as p16INK4a and Bim/BCL2L11. EZH2 inhibitors, such as tazemetostat and GSK126, have shown promising antitumor effects in some cancers that share mechanistic features with certain EBV-associated malignancies [104, 105]. Some studies have noted that EZH2 inhibition might increase viral replication, induce the lytic cycle, and enhance the potential efficacy of immunotherapies in certain cell lines [106].Therefore, the effectiveness of EZH2 inhibitors varies depending on the specific type of EBV-associated cancer and its use in EBV-related cancers is largely in clinical trials or under investigation.

DNA methylation provides a promising platform for developing epigenetics-targeted drugs to treat a wide range of diseases, including cancers, metabolic, inflammatory, and neurological disorders. Hypomethylating agents are FDA approved foundational epigenetic therapies for myelodysplastic syndromes (MDS) and acute myeloid leukemia [107]. There are three FDA-approved HMAs (Azacitidine, Decitabine, and Decitabine/Cedazuridine) used in cancer treatment [108]. DNA methylation-targeted drugs, specifically the HMAs such as azacitidine and decitabine, are being investigated for the treatment of EBV-associated malignancies. Induction of lytic infection with HMAs may sensitize tumor cells to lysis by EBV-specific cytotoxic T cells (EBV-CTLs). Preclinical data suggest that the combination of a DNA methylation inhibitor and a HDAC inhibitor has synergistic effects on gene expression in tumor cells. Therefore, HMAs can be used as part of combination therapies rather than as single agents for EBV-associated malignancies [109–111]. With significant advances in optimizing HMAs and their administration strategies, promising antitumor effects have been demonstrated in various studies. The primary mechanism showing promise in solid tumor therapy is the ability of HMAs to increase the immunologic visibility of tumor cells to the host immune system by reversing aberrant DNA methylation, thereby restoring the expression of silenced tumor suppressor genes that can be recognized by the host immune system. This immunomodulatory mechanism suggests that HMAs could be repurposed for the treatment of EBV-associated malignancies, where viral latency and oncogenesis are closely linked to DNA methylation-mediated epigenetic mechanisms. By demethylating the EBV genome, HMAs may enhance viral antigen presentation and sensitize EBV-positive tumor cells to immune-mediated clearance and immunotherapy.

Several epigenetic therapeutic strategies aim to inhibit DNA methylation in cancer cells. UHRF1 is a host protein critical for maintaining DNA methylation and is overexpressed in numerous human cancer types. EBV upregulates UHRF1 and DNMT1 to silence the expression of most viral genes through DNA methylation. This enables the virus to persist in a latent state, hidden from the host’s immune system. Inhibiting UHRF1 disrupts the viral silencing mechanism, leading to demethylation of viral promoters and re-expression of viral antigens. The re-expression of immunogenic viral proteins renders the cancer cells visible to the host’s cytotoxic T-cells. Several UHRF1 inhibitors have been developed and reported, including mitoxantrone, idarubicin, NSC232003, doxorubicin, AMSA2, MPB7, UF146, and MLD3-5 [37, 112]. DNA methylation-targeted drugs are not yet approved for EBV-related diseases, but their potent epigenetic-modifying effects make them a promising component of ongoing research and clinical trials for various EBV-associated cancers. Mitoxantrone is FDA-approved and remains a critical, though highly specialized, therapy in oncology. Doxorubicin is FDA-approved and a first-line treatment for a vast range of cancers, including leukemias, lymphomas, breast, bladder, ovarian, and lung carcinomas. Doxorubicin is primarily known as a cytotoxic agent, its secondary role as a potent epigenetic modifier has become a major focus of modern precision oncology. Doxorubicin acts on the DNA methylation machinery to restore viral and tumor suppressor genes. Doxorubicin activates transcription from promoters of the BZLF1 and BRLF1 genes and is primarily used within multi-agent chemotherapy regimens for EBV-positive hematologic cancers. Clinical trials for next-generation variants of Doxorubicin such as Faridoxorubicin (AVA6000) have shown high safety [113].

Recent research has revealed a complex interplay between the EBV life cycle and the host’s RNA methylation machinery, specifically N6-methyladenosine (m6A). The methyltransferase METTL14 is critical for EBV gene expression during latent infection and virus-associated tumorigenesis. The latent oncoprotein EBNA3C activates the transcription of METTL14 and directly interacts with METTL14 to promote its stability [114]. The host m6A-binding protein YTHDF1 suppresses EBV infection and replication. YTHDF1 mediates RNA decay by recruiting RNA degradation complexes, including DDX17, ZAP, and DCP2, thereby post-transcriptionally downregulating the expression of EBV lytic genes [115]. Additional studies have identified YTHDF2 and ALKBH5 as key regulators of EBV lytic cycle and antiviral immune responses. YTHDF2 functions as an antiviral restriction factor by promoting the degradation of m6A-modified viral transcripts, whereas ALKBH5 contributes to the regulation of interferon signaling pathways during EBV reactivation [57–59]. Although, RNA demethylases represent an exciting and emerging area of basic research in EBV biology and m6A machinery are increasingly recognized as potential therapeutic targets, but no clinically approved epitranscriptomic therapies are currently available for EBV-associated diseases [57, 58]. Future strategies designed to modulate RNA methylation pathways may complement existing epigenetic therapies targeting viral latency. Understanding these mechanisms suggests that manipulating EBV epitranscriptome reprogramming pathways could offer novel strategies for treating EBV-associated cancers.

### Epigenome editing and targeted reactivation of EBV

Beyond conventional epigenetic drugs, recent advances in genome and epigenome engineering have introduced epigenome editing as highly targeted strategies for manipulating the EBV latent–lytic switch. A growing body of evidence suggests that direct targeting of the viral episome may provide greater specificity than pharmacological modulation of host epigenetic enzymes. Recent reviews have highlighted the therapeutic potential of EBV-directed epigenome editing approaches that selectively reactivate latent viral genomes in EBV-associated malignancies [116].

CRISPR-based transcriptional activation systems have been developed to directly target the BZLF1 (ZTA) promoter, the master regulator of EBV lytic reactivation. Using catalytically inactive Cas9 (dCas9) fused to transcriptional activation domains, targeted activation of the ZTA promoter induced robust lytic reactivation in Bl, GC, and NPC models. Importantly, dCas9-mediated viral reactivation sensitized EBV-positive tumor cells to ganciclovir-mediated cytotoxicity, resulting in selective elimination of infected cells [117].

Similarly, transcription activator-like effector (TALE)-based synthetic transcription factors have been engineered to specifically activate the BZLF1 promoter. Delivery of a BZLF1-targeting activator induced EBV lytic gene expression and significantly suppressed tumor growth in preclinical models. Combination treatment with ganciclovir further enhanced antitumor efficacy. These emerging approaches represent a new generation of precision epigenetic therapies that directly manipulate the viral latent–lytic switch and may complement existing epigenetic drug-based strategies for EBV-associated malignancies [118].

## Conclusion

EBV infection is associated with a wide spectrum of disorders, ranging from the common, self-limiting infectious mononucleosis to various aggressive malignancies and a growing list of autoimmune diseases. EBV adopts different gene expression profiles to persist in host cells, with viral gene expression controlled by epigenetic mechanisms. Understanding the virus-host epigenetic interplay offers new opportunities for developing therapeutic interventions that target epigenetic regulators in EBV-associated diseases. Many small-molecule inhibitors targeting epigenetic-modifying enzymes have been identified, and novel technologies and mechanisms are actively being applied to optimize their physical properties and reduce cytotoxic effects, thereby accelerating their use in clinical trials. Furthermore, the reversibility of many epigenetic modifications makes them attractive targets for novel therapeutic interventions, and drugs targeting these mechanisms are already used in the treatment of certain cancers and other diseases. Combining epigenetic modulators with existing antivirals or immunotherapies is a major area of current research aiming at achieving functional cures for persistent viral infections and their associated diseases. In this regard, further progress may help to reduce the burden of EBV-associated cancer.

## Data Availability

No datasets were generated or analysed during the current study.
